# A new solvate of epalerstat, a drug for diabetic neuropathy

**DOI:** 10.1107/S2056989017010751

**Published:** 2017-07-28

**Authors:** Okky Dwichandra Putra, Daiki Umeda, Kaori Fukuzawa, Mihoko Gunji, Etsuo Yonemochi

**Affiliations:** a School of Pharmacy and Pharmaceutical Sciences, Hoshi University, 2-4-41, Ebara, Shinagawa, Tokyo 145-8501, Japan

**Keywords:** crystal structure, epalerstat, acetone, monosolvate, isotypic, hydrogen bonding

## Abstract

Epalerstat {systematic name: (5*Z*)-5-[(2*E*)-2-methyl-3-phenyl­prop-2-en-1-yl­idene]-4-oxo-2-sulfanyl­idene-1,3-thia­zolidine-3-acetic acid} crystallized as an acetone monosolvate, C_15_H_13_NO_3_S_2_·C_3_H_6_O. In the epalerstat mol­ecule, the methyl­propyl­enediene moiety is inclined to the phenyl ring and the five-membered rhodamine ring by 21.4 (4) and 4.7 (4)°, respectively. In addition, the acetic acid moiety is found to be almost normal to the rhodamine ring, making a dihedral angle of 85.1 (2)°. In the crystal, a pair of O—H⋯O hydrogen bonds between the carb­oxy­lic acid groups of epalerstat mol­ecules form inversion dimers with an *R*_2_^2^(8) loop. The dimers are linked by pairs of C—H⋯O hydrogen bonds, enclosing *R*_2_^2^(20) loops, forming chains propagating along the [101] direction. In addition, the acetone mol­ecules are linked to the chain by a C—H⋯O hydrogen bond. Epalerstat acetone monosolvate was found to be isotypic with epalerstat tertra­hydro­furan solvate [Umeda *et al.* (2017 ▸). *Acta Cryst*. E**73**, 941–944].

## Abstract 2

The title compound, epalerstat acetone monosolvate, is isotypic with the previously reported tetra­hydro­furan solvate.

## Chemical context   

Investigation of solid forms of pharmaceuticals has attracted a great deal of attention as different crystal forms may imply different physicochemical properties (Putra *et al.*, 2016*a* ▸,*b* ▸). Moreover, pharmaceutical processing stages during manufacturing, such as crystallization, can lead to the unexpected occurrence of new crystalline phases (Putra *et al.*, 2016*c* ▸). One of the important classes of pharmaceutical solids that can occur during crystallization is solvates. Solvates are defined as multi-component crystalline systems in which solvent mol­ecules are included within the crystal structure in either a stoichiometric or non-stoichiometric manner (Griesser, 2006 ▸). It has been estimated statistically that around 33% of organic compounds have the ability to form solvates with organic solvents (Clarke *et al.*, 2010 ▸).
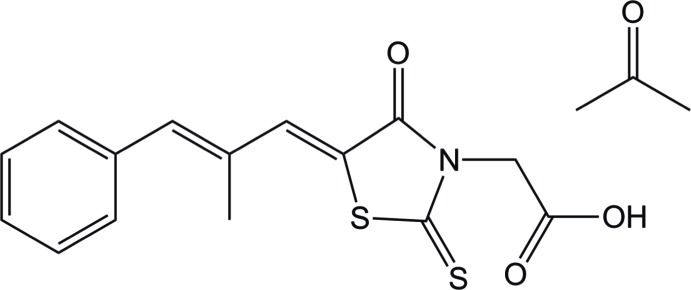


Herein, we report on the crystal structure of a new solvate form of epalerstat, namely epalerstat acetone monosolvate. Epalerstat [systematic name: (5*Z*)-5-[(2*E*)-2-methyl-3-phenyl­prop-2-en-1-yl­idene]-4-oxo-2-sulfanyl­idene-1,3-thia­zol­idine-3-acetic acid), is an aldose reductase inhibitor and is used for the treatment of diabetic neuropathy, a complication symptom in diabetes mellitus (Miyamoto, 2002 ▸). Pharmacologically, epalerstat acts to inhibit the synthesis of sorbitol from glucose (Ramirez & Borja, 2008 ▸). The abundant occurrences of solvates in epalerstat itself is not surprising because of the imbalance between the hydrogen-bond donors and acceptors in its mol­ecular structure. Previously, the crystal structures of the methanol mono- and disolvate (Igarashi *et al.*, 2015 ▸; Nagase *et al.*, 2016 ▸), the ethanol monosolvate (Ishida *et al.*, 1989 ▸, 1990 ▸), the di­methyl­formamide monosolvate (Putra *et al.*, 2017 ▸), the di­methyl­sulfoxide disolvate (Putra *et al.*, 2017 ▸) and the tetra­hydro­furan monosolvate (Umeda *et al.*, 2017 ▸) have been reported.

## Structural commentary   

**Figure 1:**
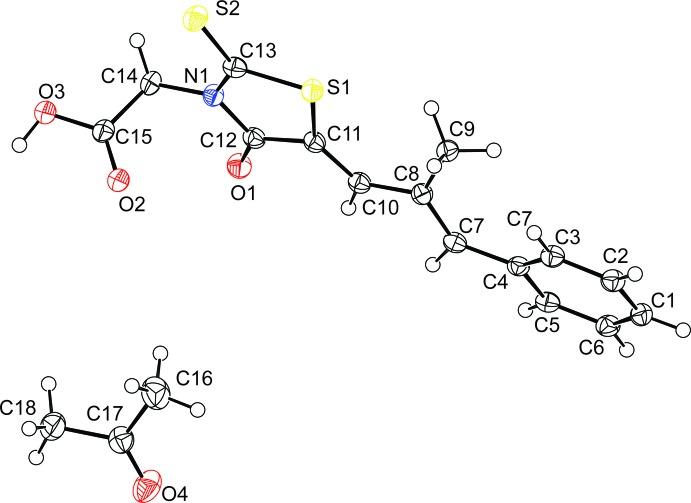
The mol­ecular structure of epalerstat acetone monosolvate, with the atom labelling and displacement ellipsoids drawn at the 50% probability level.

The mol­ecular structure of epalerstat acetone monosolvate is illustrated in Fig. 1 ▸. The values of the bond distances, bond angles and dihedral angles are normal according the *Mogul* geometry check within the CSD software (Bruno *et al.*, 2004 ▸; Groom *et al.*, 2016 ▸). The mean plane of the methyl­propyl­enediene (C7–C10) moiety is inclined to the phenyl ring (C1–C6) and the five-membered rhodamine ring (S1/S2/O1/N1/C11–C13) by 21.4 (4) and 4.7 (4)°, respectively. The mean plane of the acetic acid moiety (O2/O3/C14/C15) is almost normal to the rhodamine ring, making a dihedral angle of 85.1 (2) °.

## Supra­molecular features   

**Figure 2:**
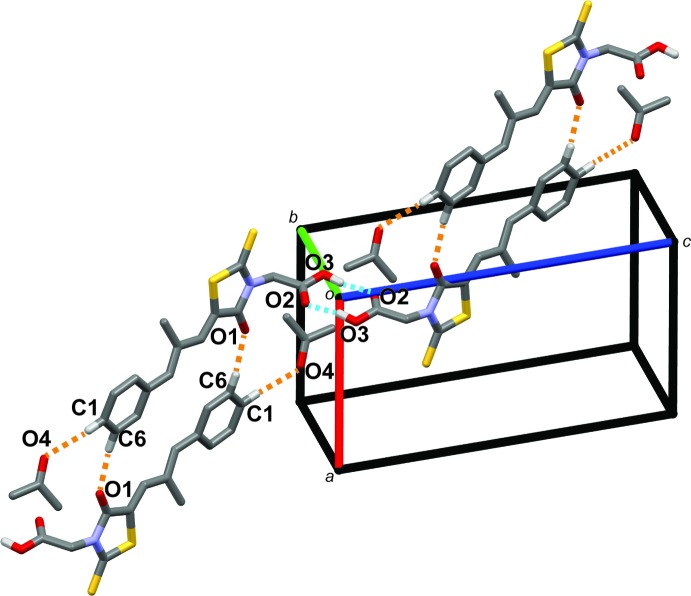
A view along the *b* axis of the crystal packing of the title compound. Blue and orange dashed lines represent O—H⋯O and C—H⋯O hydrogen bonds, respectively. Only H atoms involved in these inter­actions have been included.

In the crystal, the epalerstat mol­ecule is connected to two adjacent epalerstat mol­ecules and one solvent mol­ecule *via* both conventional and non-conventional hydrogen bonds. The details of the hydrogen bonds and hydrogen bonding architecture are listed and presented in Table 1 ▸ and Fig. 2 ▸, respectively. A pair of O3—H3*A*⋯O2^ii^ hydrogen bonds is observed between two carb­oxy­lic acid moieties forming an inversion dimer with an 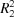
(8) loop. This dimer is linked to adjacent dimers by a pair of C6—H6⋯O1^ii^ hydrogen bonds, which enclose 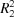
(20) loops, and form chains along direction [101]. In addition, acetone mol­ecules are linked to the chain by a C1—H1⋯O4^iii^ hydrogen bond (Table 1 ▸ and Fig. 2 ▸).

### Discussion   

**Figure 3:**
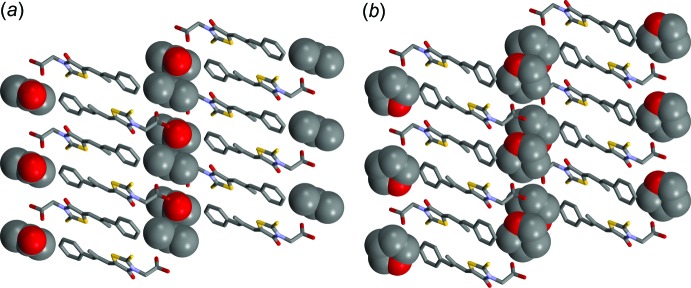
The packing view along the *b* axis of (*a*) epalerstat acetone monosolvate and (*b*) epalerstat tetra­hydro­furan monosolvate shows the isostructurality between the two solvates. H atoms have been omitted for clarity, and the epalerstat mol­ecules and the solvent mol­ecules are drawn as capped sticks and spacefill models, respectively.

Inter­estingly, the new solvate reported here is isotypic with epalerstat tetra­hydro­furan monosolvate (Umeda *et al.*, 2017 ▸). Both solvates crystallize in the triclinic system with the same space group, *P* 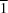. As illustrated in Fig. 3 ▸, they have a similar mol­ecular arrangement and the solvent mol­ecules are located in similar pockets in the unit cell. The unit cell similarity index (*Π*) and the mean elongation (*∊*) values were calculated (Fábián & Kálmán, 1999 ▸) and found to be *Π* = 0.0016 and *∊* = 0.0005. As the *Π* and *∊* values are nearly zero, epalerstat acetone monosolvate and tetra­hydro­furan monosolvate have isostructural crystals. The solvent-occupied spaces, in which the solvent mol­ecules were deleted from the crystal structure, and the voids were calculated using the contact surface method with probe radius and approximate grid spacing set equal to 1.2 and 0.7 Å, respectively (Putra *et al.*, 2016**d* ▸;* Macrae *et al.*, 2008 ▸). The solvent occupied spaces for the acetone and tetra­hydro­furan solvates are 199.86 and 221.89 Å^3^, respectively. As expected, the larger occupied space in epalerstat tetra­hydro­furan solvate corresponds to the larger solvent mol­ecule. Inter­estingly, both solvents occupy nearly the same percentage of the total volume of the unit cell; the acetone and tetra­hydro­furan mol­ecules occupy 22.2 and 23.8%, respectively.

## Database survey   

A search of the Cambridge Structural Database (CSD, V5.38, last update July 2017; Groom *et al.*, 2016 ▸) for epalerstat yielded 16 hits. They include the ethanol monosolvate (Ishida *et al.*, 1989 ▸, 1990 ▸), the methanol monosolvate (Igarashi *et al.*, 2015 ▸), the methanol disolvate (Nagase *et al.*, 2016 ▸), the di­methyl­formamide monosolvate (Putra *et al.*, 2017 ▸), the di­methyl­sulfoxide disolvate (Putra *et al.*, 2017 ▸), the tetra­hydro­furan monosolvate (Umeda *et al.*, 2017 ▸), Form I: triclinic, *P* 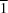 (Igarashi *et al.*, 2013 ▸; Swapna *et al.*, 2016 ▸), Form II: monoclinic, *C*2/*c* (Swapna *et al.*, 2016 ▸), Form III: monoclinic, *P*2_1_/*n* (Swapna *et al.*, 2016 ▸), the co-crystal with caffeine (Putra *et al.*, 2017 ▸), a series of salt co-crystals with cytosine (Swapna & Nangia, 2017 ▸) and the *Z,Z* isomer (Swapna *et al.*, 2016 ▸).

## Synthesis and crystallization   

Epalerstat Form I (700 mg) was dissolved in 10 ml acetone and the clear solution was then kept for three days at room temperature. Epalerstat acetone monosolvate appeared concomitantly with epalerstat Form I and they could be distinguished visually based on their crystal habit. In this case, the title compound, epalerstat acetone monosolvate, and Form I appeared as yellow blocks and orange needle-like crystals, respectively.

## Refinement details   

Crystal data, data collection and structure refinement details are summarized in Table 2 ▸. The hydrogen atom attached to an oxygen atom was located in a difference-Fourier map and freely refined. The C-bound H atoms were included in calculated positions and treated using riding model: C—H = 0.9–1.0 Å with *U*_iso_(H) = 1.5*U*_iso_(C-meth­yl) and 1.2*U*_iso_(C) for other H atoms. Initially the site occupancy factor of the acetone mol­ecule was refined and determined to be 1.005 (4). In the final cycles of refinement the occupancy of the acetone mol­ecule was fixed at 1.

## Supplementary Material

Crystal structure: contains datablock(s) I, Global. DOI: 10.1107/S2056989017010751/su5385sup1.cif

Structure factors: contains datablock(s) I. DOI: 10.1107/S2056989017010751/su5385Isup2.hkl

Supporting information file. DOI: 10.1107/S2056989017010751/su5385Isup3.cml

CCDC reference: 1563705

Additional supporting information:  crystallographic information; 3D view; checkCIF report

## Figures and Tables

**Table 1:** Hydrogen-bond geometry (Å, °)

*D*—H⋯*A*	*D*—H	H⋯*A*	*D*⋯*A*	*D*—H⋯*A*
O3—H3*A*⋯O2^i^	0.91 (3)	1.75 (3)	2.645 (2)	171 (3)
C6—H6⋯O1^ii^	0.95	2.50	3.440 (3)	168
C1—H1⋯O4^iii^	0.95	2.58	3.525 (3)	171

Symmetry codes: (i) 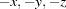; (ii) 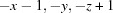; (iii) 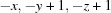.

**Table 2:** Experimental details

Crystal data	
Chemical formula	C_15_H_13_NO_3_S_2_·C_3_H_6_O
*M*_r_	377.46
Crystal system, space group	Triclinic, *P* 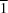
Temperature (K)	93
*a*, *b*, *c* (Å)	7.9623 (1), 8.1806 (2), 15.6919 (3)
α, β, γ (°)	97.852 (7), 99.837 (7), 113.206 (8)
*V* (Å^3^)	901.83 (6)
*Z*	2
Radiation type	Cu *K*α
μ (mm^−1^)	2.87
Crystal size (mm)	0.35 × 0.24 × 0.10

Data collection	
Diffractometer	Rigaku R-AXIS RAPID II
Absorption correction	Multi-scan (*ABSCOR*; Higashi, 1995 ▸)
*T*_min_, *T*_max_	0.378, 0.750
No. of measured, independent and observed [*I* > 2σ(*I*)] reflections	10593, 3235, 2790
*R*_int_	0.045
(sin θ/λ)_max_ (Å^−1^)	0.602

Refinement	
*R*[*F* ^2^ > 2σ(*F* ^2^)], *wR*(*F* ^2^), *S*	0.050, 0.137, 1.11
No. of reflections	3235
No. of parameters	233
H-atom treatment	H atoms treated by a mixture of independent and constrained refinement
Δρ_max_, Δρ_min_ (e Å^−3^)	0.73, −0.30

Computer programs: *PROCESS-AUTO* (Rigaku, 1998 ▸), *SHELXS2014* (Sheldrick, 2008 ▸), *Mercury* (Macrae *et al.*, 2008 ▸), *SHELXL2016* (Sheldrick, 2015 ▸) and *PLATON* (Spek, 2009 ▸).

## supplementary crystallographic information

### Crystal data

**Table:**

C_15_H_13_NO_3_S_2_·C_3_H_6_O	*Z* = 2
*M**_r_* = 377.46	*F*(000) = 396
Triclinic, *P*1	*D*_x_ = 1.390 Mg m^−^^3^
*a* = 7.9623 (1) Å	Cu *K*α radiation, λ = 1.54187 Å
*b* = 8.1806 (2) Å	Cell parameters from 10593 reflections
*c* = 15.6919 (3) Å	θ = 5.9–68.2°
α = 97.852 (7)°	µ = 2.87 mm^−^^1^
β = 99.837 (7)°	*T* = 93 K
γ = 113.206 (8)°	Block, yellow
*V* = 901.83 (6) Å^3^	0.35 × 0.24 × 0.10 mm

### Data collection

**Table:**

Rigaku R-AXIS RAPID II diffractometer	3235 independent reflections
Radiation source: rotating anode X-ray, RIGAKU	2790 reflections with *I* > 2σ(*I*)
Detector resolution: 10.0 pixels mm^-1^	*R*_int_ = 0.045
ω scan	θ_max_ = 68.2°, θ_min_ = 5.9°
Absorption correction: multi-scan (ABSCOR; Higashi, 1995)	*h* = −9→9
*T*_min_ = 0.378, *T*_max_ = 0.750	*k* = −9→9
10593 measured reflections	*l* = −18→18

### Refinement

**Table:**

Refinement on *F*^2^	Primary atom site location: structure-invariant direct methods
Least-squares matrix: full	Secondary atom site location: difference Fourier map
*R*[*F*^2^ > 2σ(*F*^2^)] = 0.050	Hydrogen site location: mixed
*wR*(*F*^2^) = 0.137	H atoms treated by a mixture of independent and constrained refinement
*S* = 1.11	*w* = 1/[σ^2^(*F*_o_^2^) + (0.0886*P*)^2^] where *P* = (*F*_o_^2^ + 2*F*_c_^2^)/3
3235 reflections	(Δ/σ)_max_ < 0.001
233 parameters	Δρ_max_ = 0.73 e Å^−^^3^
0 restraints	Δρ_min_ = −0.30 e Å^−^^3^

### Special details

**Table:**

Geometry. Bond distances, angles etc. have been calculated using the rounded fractional coordinates. All su's are estimated from the variances of the (full) variance-covariance matrix. The cell esds are taken into account in the estimation of distances, angles and torsion angles

### Fractional atomic coordinates and isotropic or equivalent isotropic displacement parameters (Å^2^)

**Table:**

	*x*	*y*	*z*	*U*_iso_*/*U*_eq_	
S1	0.32326 (7)	0.22776 (7)	0.40775 (3)	0.0232 (2)	
S2	0.53005 (7)	0.15013 (8)	0.27814 (4)	0.0290 (2)	
O1	−0.15383 (19)	−0.1417 (2)	0.26694 (9)	0.0270 (5)	
O2	0.0245 (2)	0.0465 (2)	0.10793 (9)	0.0257 (5)	
O3	0.0821 (2)	−0.1687 (2)	0.02943 (10)	0.0304 (5)	
N1	0.1578 (2)	−0.0247 (2)	0.26497 (11)	0.0214 (5)	
C1	−0.2491 (3)	0.5295 (3)	0.76805 (14)	0.0256 (7)	
C2	−0.0768 (3)	0.6025 (3)	0.74641 (14)	0.0252 (7)	
C3	−0.0449 (3)	0.5163 (3)	0.67266 (13)	0.0232 (6)	
C4	−0.1877 (3)	0.3532 (3)	0.61713 (14)	0.0214 (6)	
O4	0.3463 (2)	0.3068 (2)	0.03521 (11)	0.0438 (6)	
C5	−0.3632 (3)	0.2835 (3)	0.63920 (14)	0.0235 (6)	
C6	−0.3931 (3)	0.3700 (3)	0.71326 (14)	0.0252 (7)	
C7	−0.1685 (3)	0.2516 (3)	0.53833 (13)	0.0216 (6)	
C8	−0.0128 (3)	0.2508 (3)	0.51367 (13)	0.0215 (6)	
C9	0.1858 (3)	0.3596 (3)	0.56879 (14)	0.0242 (6)	
C10	−0.0467 (3)	0.1284 (3)	0.43048 (13)	0.0218 (6)	
C11	0.0769 (3)	0.1067 (3)	0.38588 (13)	0.0211 (6)	
C12	0.0075 (3)	−0.0322 (3)	0.30199 (13)	0.0220 (7)	
C13	0.3343 (3)	0.1078 (3)	0.30963 (14)	0.0226 (6)	
C14	0.1251 (3)	−0.1486 (3)	0.18203 (13)	0.0234 (6)	
C15	0.0718 (3)	−0.0778 (3)	0.10282 (14)	0.0230 (6)	
C16	0.6721 (4)	0.3855 (4)	0.08636 (16)	0.0463 (9)	
C17	0.4865 (3)	0.2987 (3)	0.01874 (15)	0.0306 (7)	
C18	0.4837 (4)	0.2023 (4)	−0.07037 (16)	0.0397 (8)	
H1	−0.26853	0.58768	0.81968	0.0310*	
H2	0.02089	0.71326	0.78271	0.0300*	
H3	0.07467	0.56789	0.65942	0.0280*	
H3A	0.034 (4)	−0.130 (4)	−0.016 (2)	0.066 (10)*	
H5	−0.46298	0.17474	0.60237	0.0280*	
H6	−0.51254	0.32025	0.72678	0.0300*	
H7	−0.28463	0.17230	0.49709	0.0260*	
H9A	0.24324	0.47371	0.54951	0.0360*	
H9B	0.25903	0.28868	0.56141	0.0360*	
H9C	0.18490	0.38731	0.63143	0.0360*	
H10	−0.17563	0.05124	0.40288	0.0260*	
H14A	0.02271	−0.26889	0.17941	0.0280*	
H14B	0.24067	−0.16584	0.17988	0.0280*	
H16A	0.66246	0.45979	0.13819	0.0690*	
H16B	0.76964	0.46315	0.06051	0.0690*	
H16C	0.70570	0.29014	0.10460	0.0690*	
H18A	0.35396	0.14160	−0.10715	0.0590*	
H18B	0.53261	0.11105	−0.06269	0.0590*	
H18C	0.56266	0.29111	−0.09945	0.0590*	

### Atomic displacement parameters (Å^2^)

**Table:**

	*U*^11^	*U*^22^	*U*^33^	*U*^12^	*U*^13^	*U*^23^
S1	0.0185 (3)	0.0257 (3)	0.0223 (3)	0.0072 (2)	0.0049 (2)	0.0033 (2)
S2	0.0207 (3)	0.0327 (4)	0.0313 (3)	0.0088 (3)	0.0094 (2)	0.0041 (3)
O1	0.0193 (8)	0.0290 (9)	0.0267 (8)	0.0060 (7)	0.0039 (6)	0.0033 (7)
O2	0.0287 (8)	0.0273 (9)	0.0227 (8)	0.0147 (7)	0.0057 (6)	0.0029 (6)
O3	0.0355 (9)	0.0386 (10)	0.0221 (8)	0.0233 (8)	0.0047 (7)	0.0020 (7)
N1	0.0205 (9)	0.0223 (10)	0.0204 (9)	0.0082 (8)	0.0056 (7)	0.0036 (7)
C1	0.0304 (12)	0.0290 (13)	0.0238 (11)	0.0168 (10)	0.0099 (9)	0.0091 (9)
C2	0.0251 (11)	0.0242 (12)	0.0267 (11)	0.0115 (9)	0.0049 (9)	0.0056 (9)
C3	0.0196 (10)	0.0233 (12)	0.0266 (11)	0.0081 (9)	0.0064 (9)	0.0074 (9)
C4	0.0221 (11)	0.0232 (11)	0.0232 (11)	0.0123 (9)	0.0066 (9)	0.0091 (9)
O4	0.0331 (10)	0.0508 (11)	0.0447 (11)	0.0144 (8)	0.0180 (8)	0.0027 (9)
C5	0.0192 (10)	0.0248 (12)	0.0268 (11)	0.0099 (9)	0.0038 (9)	0.0073 (9)
C6	0.0205 (11)	0.0300 (13)	0.0281 (11)	0.0119 (9)	0.0082 (9)	0.0096 (10)
C7	0.0208 (11)	0.0199 (11)	0.0238 (11)	0.0083 (9)	0.0037 (9)	0.0073 (9)
C8	0.0231 (11)	0.0223 (11)	0.0217 (10)	0.0106 (9)	0.0074 (9)	0.0079 (9)
C9	0.0213 (11)	0.0293 (12)	0.0227 (10)	0.0122 (9)	0.0054 (9)	0.0040 (9)
C10	0.0205 (10)	0.0220 (11)	0.0232 (11)	0.0085 (9)	0.0052 (9)	0.0081 (9)
C11	0.0211 (11)	0.0212 (11)	0.0212 (10)	0.0085 (9)	0.0051 (8)	0.0077 (9)
C12	0.0216 (11)	0.0236 (12)	0.0226 (11)	0.0101 (9)	0.0056 (9)	0.0091 (9)
C13	0.0197 (11)	0.0227 (11)	0.0260 (11)	0.0089 (9)	0.0051 (9)	0.0082 (9)
C14	0.0251 (11)	0.0215 (11)	0.0230 (11)	0.0095 (9)	0.0084 (9)	0.0012 (9)
C15	0.0146 (10)	0.0261 (12)	0.0234 (11)	0.0058 (9)	0.0040 (8)	0.0000 (9)
C16	0.0394 (15)	0.0540 (18)	0.0356 (14)	0.0151 (13)	0.0027 (12)	0.0022 (13)
C17	0.0282 (13)	0.0318 (13)	0.0308 (12)	0.0100 (10)	0.0100 (10)	0.0089 (10)
C18	0.0330 (13)	0.0541 (17)	0.0317 (13)	0.0211 (12)	0.0078 (11)	0.0016 (12)

### Geometric parameters (Å, º)

**Table:**

S1—C11	1.759 (3)	C11—C12	1.476 (3)
S1—C13	1.744 (2)	C14—C15	1.507 (3)
S2—C13	1.636 (3)	C1—H1	0.9500
O1—C12	1.213 (3)	C2—H2	0.9500
O2—C15	1.213 (3)	C3—H3	0.9500
O3—C15	1.316 (3)	C5—H5	0.9500
N1—C12	1.401 (3)	C6—H6	0.9500
N1—C13	1.377 (3)	C7—H7	0.9500
N1—C14	1.451 (3)	C9—H9B	0.9800
O3—H3A	0.91 (3)	C9—H9C	0.9800
C1—C6	1.391 (3)	C9—H9A	0.9800
C1—C2	1.387 (4)	C10—H10	0.9500
C2—C3	1.386 (3)	C14—H14A	0.9900
C3—C4	1.407 (3)	C14—H14B	0.9900
C4—C5	1.410 (4)	C16—C17	1.499 (4)
C4—C7	1.459 (3)	C17—C18	1.501 (3)
O4—C17	1.212 (3)	C16—H16A	0.9800
C5—C6	1.382 (3)	C16—H16B	0.9800
C7—C8	1.363 (4)	C16—H16C	0.9800
C8—C10	1.445 (3)	C18—H18A	0.9800
C8—C9	1.501 (3)	C18—H18B	0.9800
C10—C11	1.352 (3)	C18—H18C	0.9800
			
C11—S1—C13	93.02 (11)	C4—C3—H3	120.00
C12—N1—C13	116.89 (18)	C4—C5—H5	119.00
C12—N1—C14	120.69 (18)	C6—C5—H5	119.00
C13—N1—C14	122.41 (19)	C1—C6—H6	120.00
C15—O3—H3A	107 (2)	C5—C6—H6	120.00
C2—C1—C6	119.2 (2)	C4—C7—H7	114.00
C1—C2—C3	121.0 (2)	C8—C7—H7	114.00
C2—C3—C4	120.7 (2)	C8—C9—H9A	109.00
C3—C4—C5	117.4 (2)	C8—C9—H9B	109.00
C5—C4—C7	117.6 (2)	C8—C9—H9C	109.00
C3—C4—C7	125.1 (2)	H9A—C9—H9B	109.00
C4—C5—C6	121.4 (2)	H9A—C9—H9C	109.00
C1—C6—C5	120.3 (2)	H9B—C9—H9C	109.00
C4—C7—C8	131.1 (2)	C8—C10—H10	115.00
C7—C8—C9	124.49 (19)	C11—C10—H10	115.00
C7—C8—C10	116.2 (2)	N1—C14—H14A	109.00
C9—C8—C10	119.2 (2)	N1—C14—H14B	109.00
C8—C10—C11	129.9 (2)	C15—C14—H14A	109.00
S1—C11—C12	109.41 (17)	C15—C14—H14B	109.00
C10—C11—C12	119.9 (2)	H14A—C14—H14B	108.00
S1—C11—C10	130.59 (17)	O4—C17—C16	121.6 (2)
O1—C12—C11	127.7 (2)	O4—C17—C18	121.9 (2)
N1—C12—C11	110.3 (2)	C16—C17—C18	116.5 (2)
O1—C12—N1	122.00 (19)	C17—C16—H16A	110.00
S1—C13—N1	110.27 (17)	C17—C16—H16B	109.00
S2—C13—N1	126.30 (17)	C17—C16—H16C	109.00
S1—C13—S2	123.43 (14)	H16A—C16—H16B	109.00
N1—C14—C15	111.81 (19)	H16A—C16—H16C	109.00
O2—C15—C14	123.1 (2)	H16B—C16—H16C	109.00
O3—C15—C14	111.3 (2)	C17—C18—H18A	109.00
O2—C15—O3	125.6 (2)	C17—C18—H18B	109.00
C2—C1—H1	120.00	C17—C18—H18C	110.00
C6—C1—H1	120.00	H18A—C18—H18B	109.00
C1—C2—H2	120.00	H18A—C18—H18C	109.00
C3—C2—H2	119.00	H18B—C18—H18C	109.00
C2—C3—H3	120.00		
			
C13—S1—C11—C10	−175.1 (2)	C2—C3—C4—C7	−179.8 (2)
C13—S1—C11—C12	1.91 (17)	C7—C4—C5—C6	179.3 (2)
C11—S1—C13—S2	177.60 (16)	C5—C4—C7—C8	−159.0 (2)
C11—S1—C13—N1	−2.90 (17)	C3—C4—C5—C6	−1.1 (3)
C14—N1—C13—S2	1.0 (3)	C3—C4—C7—C8	21.4 (4)
C14—N1—C12—C11	179.93 (18)	C4—C5—C6—C1	0.1 (4)
C12—N1—C13—S1	3.3 (2)	C4—C7—C8—C9	2.0 (4)
C13—N1—C12—O1	178.6 (2)	C4—C7—C8—C10	178.8 (2)
C14—N1—C12—O1	0.4 (3)	C9—C8—C10—C11	−8.4 (4)
C13—N1—C12—C11	−1.8 (3)	C7—C8—C10—C11	174.7 (2)
C13—N1—C14—C15	−93.1 (3)	C8—C10—C11—C12	178.6 (2)
C14—N1—C13—S1	−178.51 (15)	C8—C10—C11—S1	−4.7 (4)
C12—N1—C13—S2	−177.26 (17)	C10—C11—C12—O1	−3.6 (4)
C12—N1—C14—C15	85.1 (2)	C10—C11—C12—N1	176.9 (2)
C2—C1—C6—C5	1.3 (3)	S1—C11—C12—N1	−0.5 (2)
C6—C1—C2—C3	−1.8 (4)	S1—C11—C12—O1	179.0 (2)
C1—C2—C3—C4	0.8 (4)	N1—C14—C15—O2	−14.2 (3)
C2—C3—C4—C5	0.7 (3)	N1—C14—C15—O3	166.38 (19)

### Hydrogen-bond geometry (Å, º)

**Table:**

*D*—H···*A*	*D*—H	H···*A*	*D*···*A*	*D*—H···*A*
O3—H3*A*···O2^i^	0.91 (3)	1.75 (3)	2.645 (2)	171 (3)
C6—H6···O1^ii^	0.95	2.50	3.440 (3)	168
C1—H1···O4^iii^	0.95	2.58	3.525 (3)	171

Symmetry codes: (i) −*x*, −*y*, −*z*; (ii) −*x*−1, −*y*, −*z*+1; (iii) −*x*, −*y*+1, −*z*+1.
